# *En-Bloc* Kidney Transplantation From Extremely Low-Weight (0.9–5.0 kg) Pediatric Donors: A Decade of Single-Center Experience

**DOI:** 10.3389/ti.2025.14451

**Published:** 2025-05-20

**Authors:** Xianpeng Zeng, Qiuxiang Xia, Heng Li, Miao Wang, Hanying Li, Liang He, Hua Su, Chun Zhang, Zhendi Wang

**Affiliations:** ^1^ Department of Urology, Huazhong University of Science and Technology Union Hospital, Wuhan, China; ^2^ Department of Hepatobiliary Surgery, Huazhong University of Science and Technology Union Hospital, Wuhan, China; ^3^ Department of Nephrology, Huazhong University of Science and Technology Union Hospital, Wuhan, China

**Keywords:** *en-bloc*, graft failure, kidney transplantation, thrombosis, acute rejection

## Abstract

*En-bloc* kidney transplantation from low-weight pediatric donors (≤5 kg) is a challenging procedure performed only in limited transplant centers. We retrospectively analyzed the data from 42 *en-bloc* kidney transplants from donors weighing less than 5 kg between September 2014 and September 2023. The mean donor body weight was found to be 3.1 ± 1.0 kg, and the minimum weight was 0.9 kg. At a mean follow-up period of 1,481 days, the graft survival rate was 76.2% and the recipient survival rate was 100.0%. Thrombosis and acute rejection were the major complications responsible for the short-term graft loss. Male recipients were more likely to experience graft loss than female ones (P < 0.05). Recipients with long-term (>1 year) graft survival were observed to have a high prevalence (31.3%) of delayed graft function. However, they still had satisfactory long-term graft function and limited proteinuria. Continuous graft volume growth took more than 1 year to reach a stable level. Lower donor/recipient body surface area may lead to higher delayed graft function and slower estimated glomerular filtration rate recovery (P < 0.05). Kidney transplant from low-weight pediatric donors is associated with a high incidence of short-term graft loss, while long-term outcomes are generally acceptable.

## Graphical Abstract

**Figure:**
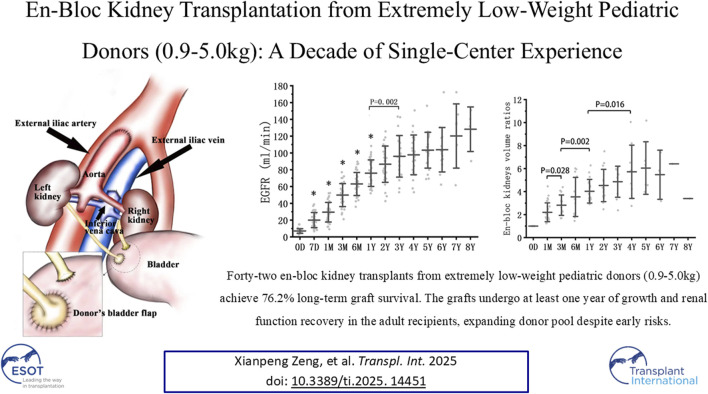

## Introduction

Pediatric *en-bloc* kidney transplantation (KTx) has been a topic of interest in the medical community for over 50 years due to its potential to alleviate the shortage of donor kidneys [1, 2]. However, due to high surgical technical requirements, along with concerns about early graft loss and hyperfiltration injury, the majority of transplant centers are still reluctant to perform it. Furthermore, recipient eligibility criteria are also a subject of debate. As a result, reports of pediatric KTx with the weight of less than 5 kg (especially <2.5 kg) are very limited, although they are considered a promising source [3]. Here, we conducted a retrospective single-center study to summarize the 42 cases of *en-bloc* KTx from pediatric donors weighing between 0.9 and 5.0 kg, and analyzed the risk factors for complications. Our study aimed to provide better clinical decisions and optimal outcomes using extremely low-weight donors for *en-bloc* KTx.

## Materials and Methods

This study included a total of 42 *en-bloc* KTx cases performed between September 2014 and September 2023. Professional human organ donation coordinators obtained written parental consent for the donation. Kidney grafts were donated by the Red Cross Society and allocated to our center by the China Organ Transplant Response System. The procedures complied with the national program of organ donation in China, the Declaration of Helsinki and the Declaration of Istanbul. This study was approved by the institutional review board of Huazhong University of Science and Technology Union Hospital (UHCT230124, Supplementary Material S1).

### Donor and Recipient Selection

All pediatric donors weighing between 0.9 and 5.0 kg conformed to the national protocol for donation after circulatory or brain death. Organ procurement was approved by the Ethics Committee of Huazhong University of Science and Technology Union Hospital. In addition to the exclusion criteria for conventional KTx, the recipients of pediatric *en-bloc* KTx were excluded if they were: (1) patients with uncontrolled hypertension or diabetes, a history of coronary heart disease or peripheral vascular disease, a hypercoagulable state, or urinary tract abnormalities; (2) patients with panel reactive antibodies >10%, a positive lymphocytotoxicity test, secondary transplantation, or lupus erythematosus; or (3) patients with a poor compliance history [4]. Recipients with low body weight were preferred. Recipients and their relatives were informed in detail about the advantages and disadvantages of *en-bloc* KTx.

### Organ Procurement

None of the livers were procured for transplantation due to the small size of the donors. A 9F or 12F sterile silicone tube without a balloon was inserted at the distal end of the abdominal aorta, with one end opening and one side opening preserved. Tube insertion depth was less than 1 cm to ensure that the cold histidine-tryptophan-ketoglutarate (HTK) solution flushing began below the level of the renal arteries. The right atrium was cut to establish the outflow to make the surgical field bloodless. The kidney surface was cooled with ice, and dissection was not started until 500 mL of HTK was perfused. Perirenal fat was kept as much as possible, and both ureters were harvested with the bladder. The inferior vena cava was dissected to the retrohepatic section and transected 1 cm above the level of the left renal vein.

### Back-Table Preparation

**FIGURE 1:**
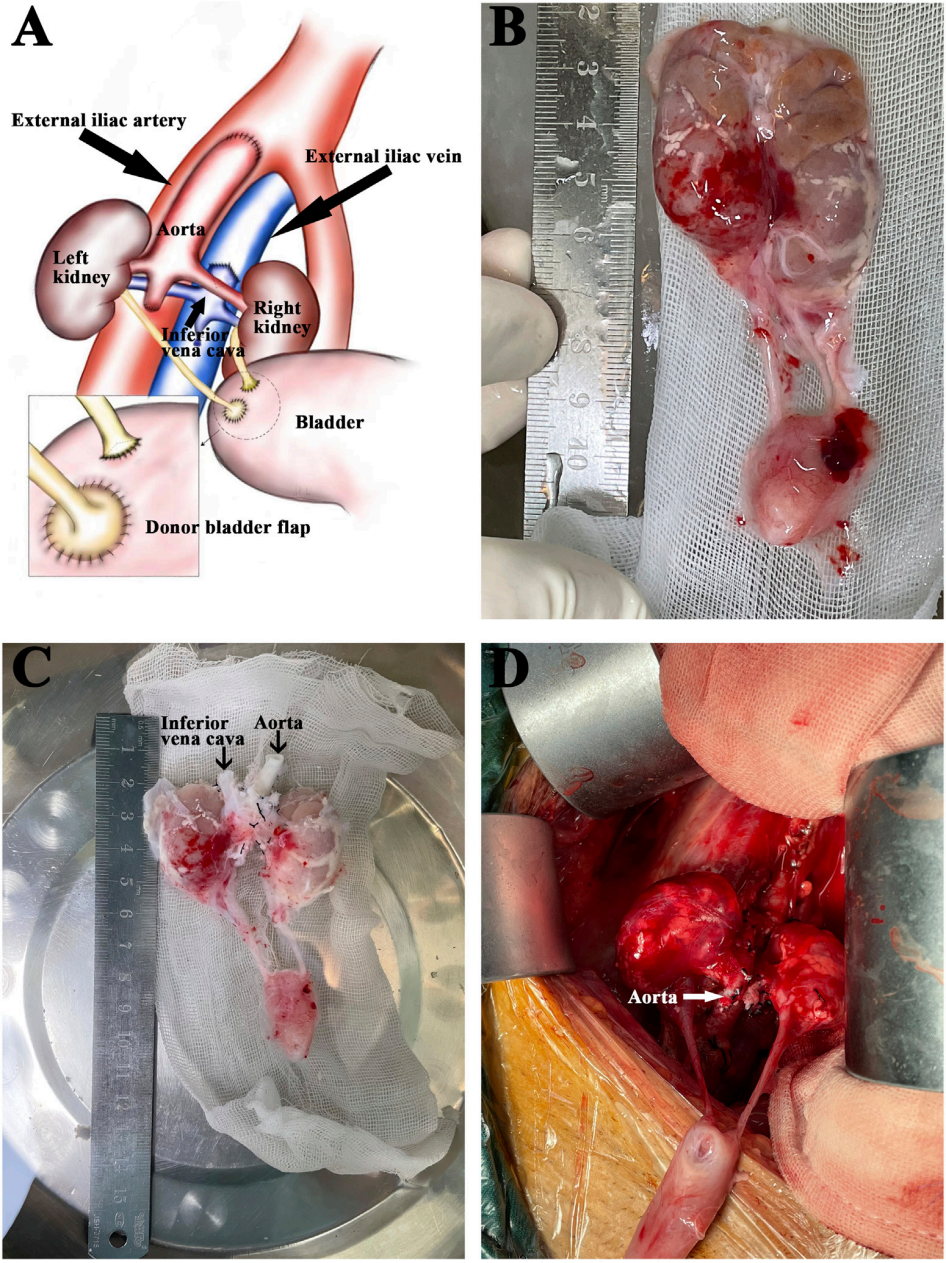
Back-table graft preparation and kidney implantation. **(A)** Diagram of the *en-bloc* renal grafts transplanted into the recipient’s right iliac. The external iliac artery was anastomosed to the donor aorta. The external iliac vein was anastomosed to the donor inferior vena cava. The recipient’s bladder received separate anastomoses of the donor’s short right ureter and the left ureter with a tiny bladder flap. **(B)** The explanted *en-bloc* renal grafts from a donor weighing 0.9 kg. **(C)** The same grafts after back-table preparation. **(D)** The same grafts after reperfusion.

Except for the renal vessels, all other aortic branches, the gonadal vein, and the tributaries of the vena cava were ligated. The tissues surrounding the renal arteries were completely preserved without exposing the arterial trunks. The bilateral adrenals were removed, and all vessels and tissue bundles were ligated away from the renal vessels. The anterior wall of the vena cava was cut open longitudinally from the proximal end. The distal aortic end was clamped, and the proximal aorta was perfused with cold HTK solution until the venous outflow fluid was clear of blood (Figures 1A-C).

### Kidney Implantation

Laterally reversed kidneys were placed extraperitoneally in the recipient’s right iliac fossa with end-to-side anastomosis to the external iliac vasculature. The proximal aorta and the V-shaped proximal vena cava of the donors were chosen for anastomosis (Figure 1D). After reperfusion, the Lich-Gregoir technique was used for implanting the internal ureter into the recipient bladder. The distal end of the ureter was transected appropriately. The external kidney was higher, so the external ureteroneocystostomy referred to an anastomosis between the donor small bladder patch and the recipient bladder. A 3F ureteral stent was placed in each ureter, with the exception of two recipients, where the stent could not be accommodated due to a thin ureteral lumen. The perirenal fat of the external kidney was fixed to the posterior muscle with one stitch before closing the incision.

### Immunosuppressive Regimen

All recipients received 1 mg/kg of rabbit anti-human thymocyte immunoglobulin (ATG) on day 0, followed by the same dosage on day 1 and half the dosage on day 2, for a cumulative dose of 2.5 mg/kg. Triple immunosuppressive therapy with tacrolimus (Tac), mycophenolate mofetil (MMF) and corticosteroids was maintained, with target trough levels for Tac. MMF doses were tapered from 1.5 g/d to 1.0 g/d at 1 month and beyond. Methylprednisolone was administered intravenously at 500 mg daily on days 0, 1, and 2, followed by oral prednisone tapering.

### Perioperative Management

No intraoperative vasodilators were routinely administered. Low molecular weight heparin (LMWH, 2000–8000 µ/day, 5–7 days) was administered subcutaneously to the first 19 subjects but not to the remaining 23 recipients. No oral antiplatelet therapy was used. During the first 14 days following KTx, the systolic blood pressure of the recipients was maintained under 140 mmHg. Graft morphology and blood flow were examined by color doppler periodically.

### Statistical Analysis

SPSS version 22 and Origin version 9 were used for statistical analyses. Mean and standard deviation were used for count data following a normal distribution, while the median was used for count data following a non-normal distribution. The Kaplan-Meier survival curve was used to evaluate graft survival. Binary logistic regression analysis was used to determine the hazard ratio of variables associated with graft loss and thrombosis. T-tests, analysis of variance and non-parametric tests were used to compare count data and P < 0.05 was considered statistically significant. The chi-square test and non-parametric test were used to compare the measurement data and P < 0.05 was considered statistically significant.

## Results

### Donor and Recipient Profiles

**FIGURE 2:**
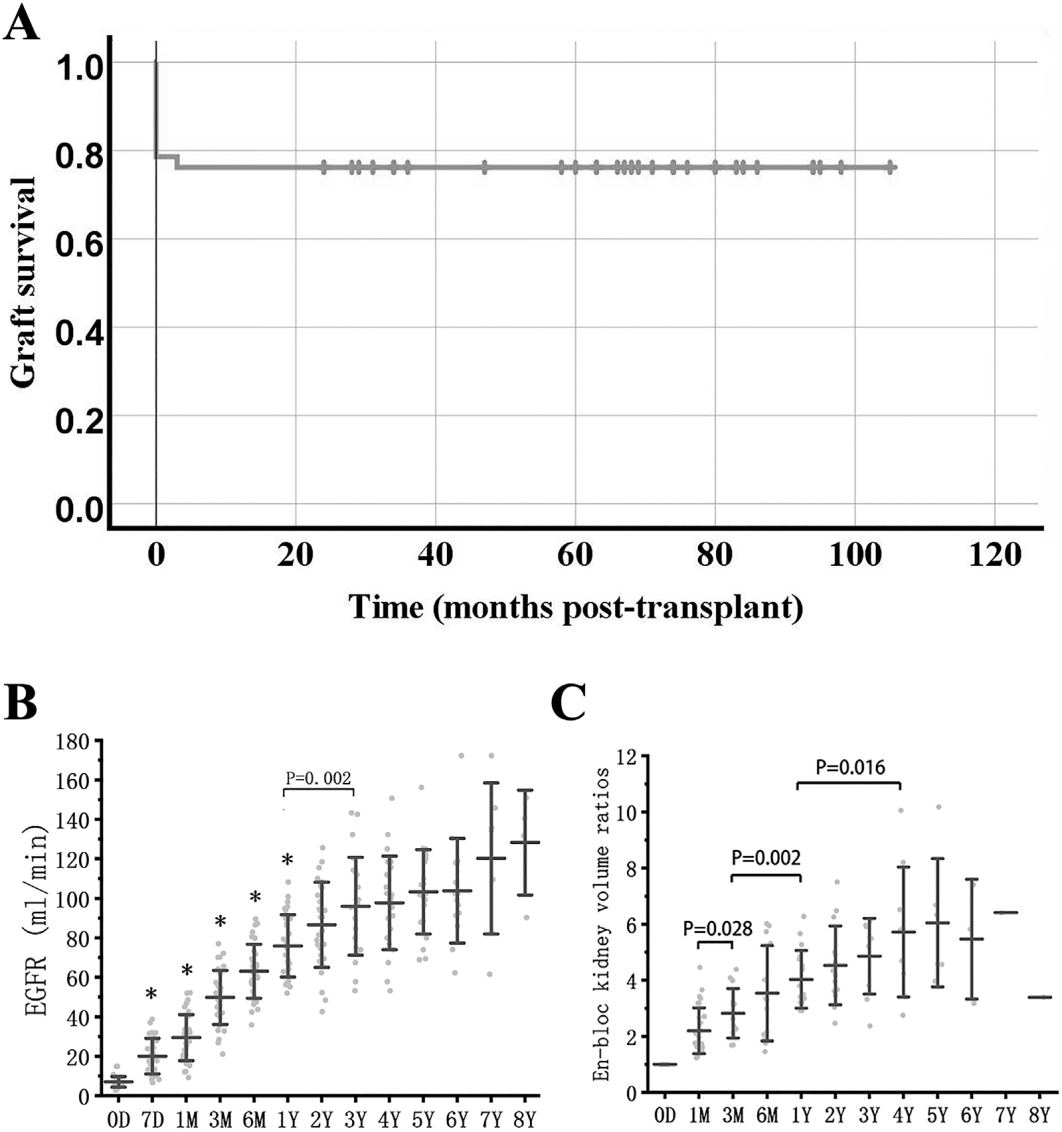
Graft survival, function and growth. **(A)** Graft survival curve in *en-bloc* kidney transplant from donors ≤5 kg. **(B)** eGFR after *en-bloc* kidney transplant. Mean and error bars are denoted within the individual plotted values. **(C)** Volume development of the grafts after transplant. Initial volume is defined as 1. * indicates a statistically significant difference relative to the previous group.

In the 42 cases of KTx, the baseline characteristics of donors and recipients are summarized in Table 1, with the minimum donor weight being 0.9 kg. Causes of death included hypoxic ischemic encephalopathy, cerebrovascular malformation, trauma, and cerebral hemispheric cyst with gliosis. There were 41 donation after circulatory death (DCD) donors and 1 donation after brain death donor, with no anencephalic donors included. All donor-recipient lymphocytotoxicity tests yielded negative results. In conjunction with previous clinical data, among the 42 recipients’ primary kidney diseases, 41 cases were glomerulonephritis without biopsy confirmation, and 1 case was IgA nephropathy. The mean follow-up time was 1,481 days (1–3,150 days). The recipient survival rate was 100%, the 1-year graft survival rate was 76.2%, and no further graft failure occurred after 1 year (Figure 2A).

**TABLE 1:** Profiles of donors and recipients for *en-bloc* kidney transplantation.

	Donors	Recipients
Age (mean)	29.4 days (4–120 days)	27.9 years (11–52 years)
Weight (mean, kg)	3.1 (0.9–5.0)	47.5 (25–64)
Gender		
Male recipients	27	16
Female recipients	15	26
Cause of death	23 hypoxic ischemic encephalopathy 16 cerebrovascular malformation 2 trauma 1 cerebral hemisphere cysticization with gliosis	
WIT (mean, min)	10.4 (6–30)	
CIT (mean, h)	11.1 (6–16)	
Primary renal disease	41 glomerulonephritis not proven by biopsy 1 IgA nephropathy	
One year graft survival		76.2%
One year recipient survival		100%

WIT, warm ischemia time; CIT, cold ischemia time.

### Graft Function and Growth

The estimated glomerular filtration rate (eGFR) of long-term survival recipients increased rapidly within 1 year after KTx, reaching 63.0 ± 13.7 mL/min at 6 months, vs. 75.7 ± 16.0 mL/min at 1 year (P = 0.001 vs. eGFR at 6 months), and continued to increase to 93.2 ± 22.8 mL/min at 3 years (P = 0.002 vs. eGFR at 1 year) (Figure 2B).

The volume of transplanted kidneys was calculated according to the Mitrou method [5]. The *en-bloc* grafts doubled in volume within the first 3 months and could reach 3-4 times the volume at 1-2 years, after which the kidney volume reached a stable level (Figure 2C).

### Graft Loss

Postoperative loss of the *en-bloc* grafts was due to arteriovenous thrombosis (6/10), acute rejection (3/10) and primary graft nonfunction (PNF) (1/10). When comparing the graft loss group with the graft survival group, baseline values were consistent, except for recipient gender (Table 2). On multivariate analysis, recipient age, donor gender, cold ischemia time (CIT) and donor/recipient body surface area (D/R BSA) ratio were not the risk factors for *en-bloc* graft loss. However, the odds ratio in female recipients compared to male recipients was 0.161 (P = 0.036; 95% CI, 0.029–0.884, Supplementary Material S2).

**TABLE 2:** Profiles of the graft survival group and the graft loss group in *en-bloc* kidney transplantation.

	Graft survival (n = 32)	Graft loss (n = 10)	P-value
Donor age (mean, d)	26.9	37.3	0.314
Donor weight (mean, kg)	3.1	3.4	0.455
Donor gender			1.000
Male recipients	20	7	
Female recipients	12	3	
Recipient age (mean, y)	27.0	30.8	0.259
Recipient weight (mean, kg)	46.7	50.0	0.193
Recipient gender			0.027
Male	9	7	
Female	23	3	
D-R BSA ratio	0.147	0.146	0.870
WIT (mean, min)	10.3	10.7	0.840
CIT (mean, h)	11.0	11.5	0.675
Mean time since the first *en-bloc* KTx (mean, d)	1,673	1,299	0.185

D/R BSA, donor/recipient body surface area; WIT, warm ischemia time; CIT, cold ischemia time; KTx, kidney transplantation.

Of the 6 recipients with thrombosis, 2 had venous thrombosis within 24 h, and 4 had arterial thrombosis between 1 and 10 days. Donor or recipient gender, age, D/R BSA, and absence of perioperative LWMH were not risk factors for thrombosis (Supplementary Material S3). Two patients with venous thrombosis experienced inadequate expansion of the venous anastomotic site following reperfusion. Despite maintaining unobstructed blood flow during the procedure, the grafts were ultimately lost within 24 h, presenting with oliguria and hematuria. The onset of arterial thrombosis is characterized by anuria, pain in the graft region or lower abdomen, and elevated blood pressure. These symptoms are analogous to arterial thrombosis in KTx from adult donors. Consequently, even in the absence of pathologic confirmation, we attributed graft loss to arterial thrombosis in four recipients.

The acute rejection episodes leading to graft loss occurred at 1–3 weeks after KTx. All three recipients did not respond to steroid and post-ATG treatment. Steroid pulse therapy was initiated immediately upon suspicion of rejection, and acute rejection was subsequently confirmed via graft biopsy. Notably, a remarkably low trough concentration of Tac was consistently observed prior to the onset of rejection.

In the case of PNF, the DCD donor succumbed to hypoxic ischemic encephalopathy. Prior to organ procurement, the donor had undergone multiple cardiopulmonary resuscitations with a warm ischemia time (WIT) of 20 min. Following transplantation, the recipient developed persistent anuria and subsequently underwent bilateral nephrectomy 3 months post-KTx.

### Other Complications

Two recipients experienced arterial stenosis after KTx. One showed a decrease in eGFR in the affected kidney 18 months after KTx, and thus underwent percutaneous transluminal angioplasty and internal stent implantation, which led to a full recovery of eGFR. The other patient, who presented with hypertension in addition to stable eGFR, had satisfactory blood pressure control after taking oral antihypertensive medicines. We found urinary leakage in 11.9% of cases, which was primarily self-limiting. One case of long-segment ureteral necrosis was treated with arterial embolization of the affected external kidney, with concern for potential surgical injury risks to the blood vessels and ureter of the internal normal kidney. Two recipients on LMWH administration underwent a second surgery for hematoma removal after KTx.

### Proteinuria

**FIGURE 3:**
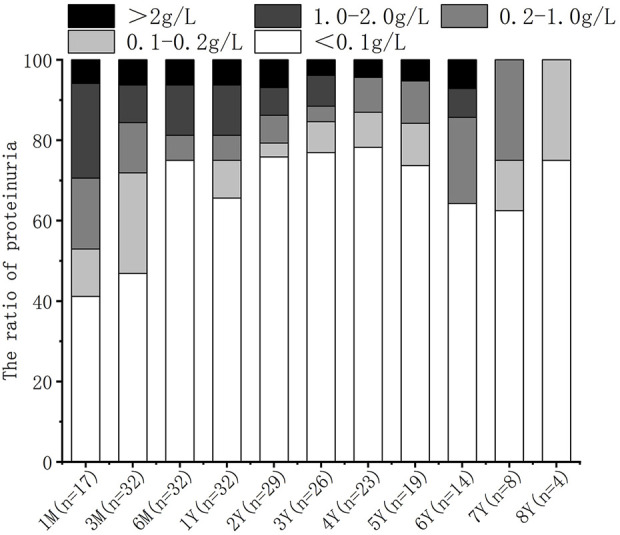
Prevalence of proteinuria after transplant.

Among the 32 recipients with long-term graft survival, proteinuria was observed in 47.1% (8/17) at 1 month, 28.1% (9/32) at 3 months, 25.0% (8/32) at 6 months and 1 year, and 20.7% (6/29) at 2 years. In order to minimize the incidence of growth disorders and nephrotoxicity, all recipients except for two patients with severe proteinuria who received treatment were not administered any medication to reduce proteinuria within 2 years of transplantation. Despite the less rigorous follow-up in this regard, the number of recipients with urine protein levels exceeding 0.2 g/L gradually decreased (Figure 3).

### Delayed Graft Function

**FIGURE 4:**
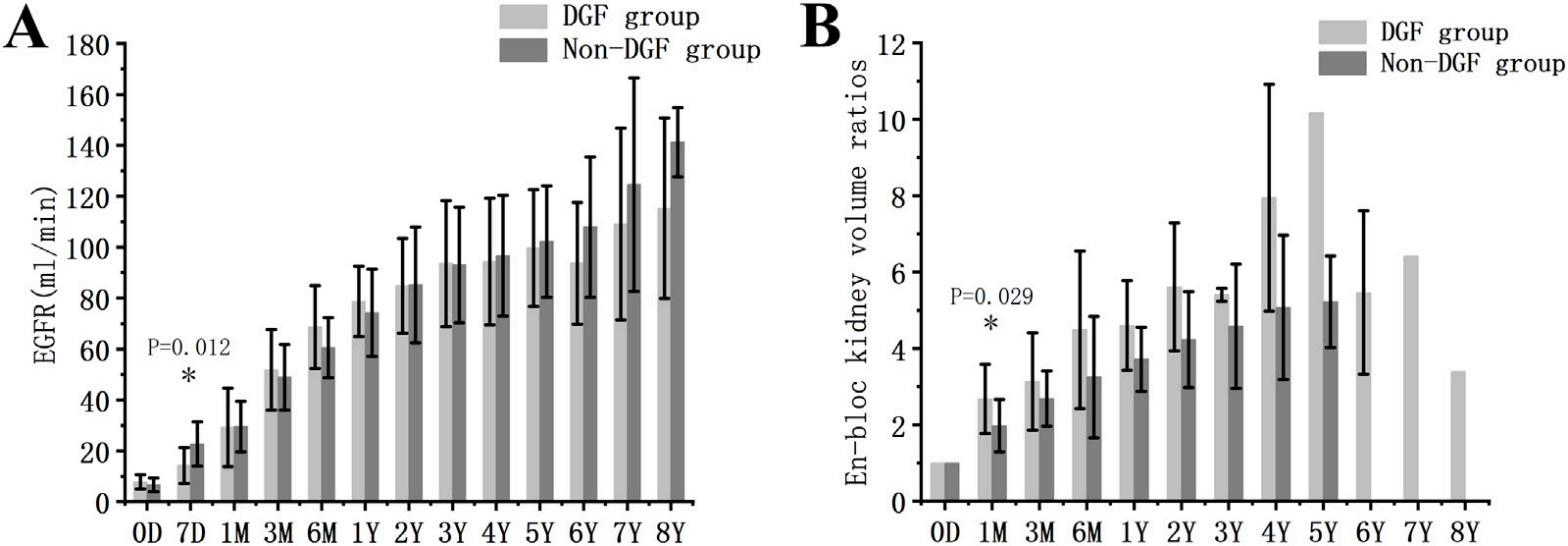
Graft function and growth in DGF and non-DGF groups. **(A)** eGFR. **(B)** Volume development of the grafts. DGF, delayed graft function.* indicates a statistically significant difference between the DGF group and the non-DGF group.

Delayed graft function (DGF) refers to the cases where dialysis is required within the first week post-KTx. Of the 32 recipients with long-term graft survival, DGF occurred in 31.3%. The DGF group had significantly lower D/R BSA compared with the non-DGF group (Supplementary Material S4). EGFR in the DGF group was significantly lower at 7 days post-KTx (P = 0.012), but not at later time points. Furthermore, the volume of transplanted kidneys in the DGF group showed a faster increase compared to the non-DGF group in the first month (P = 0.029) (Figure 4).

### Donors <2.5 kg vs. Donors >=2.5 kg

**FIGURE 5:**
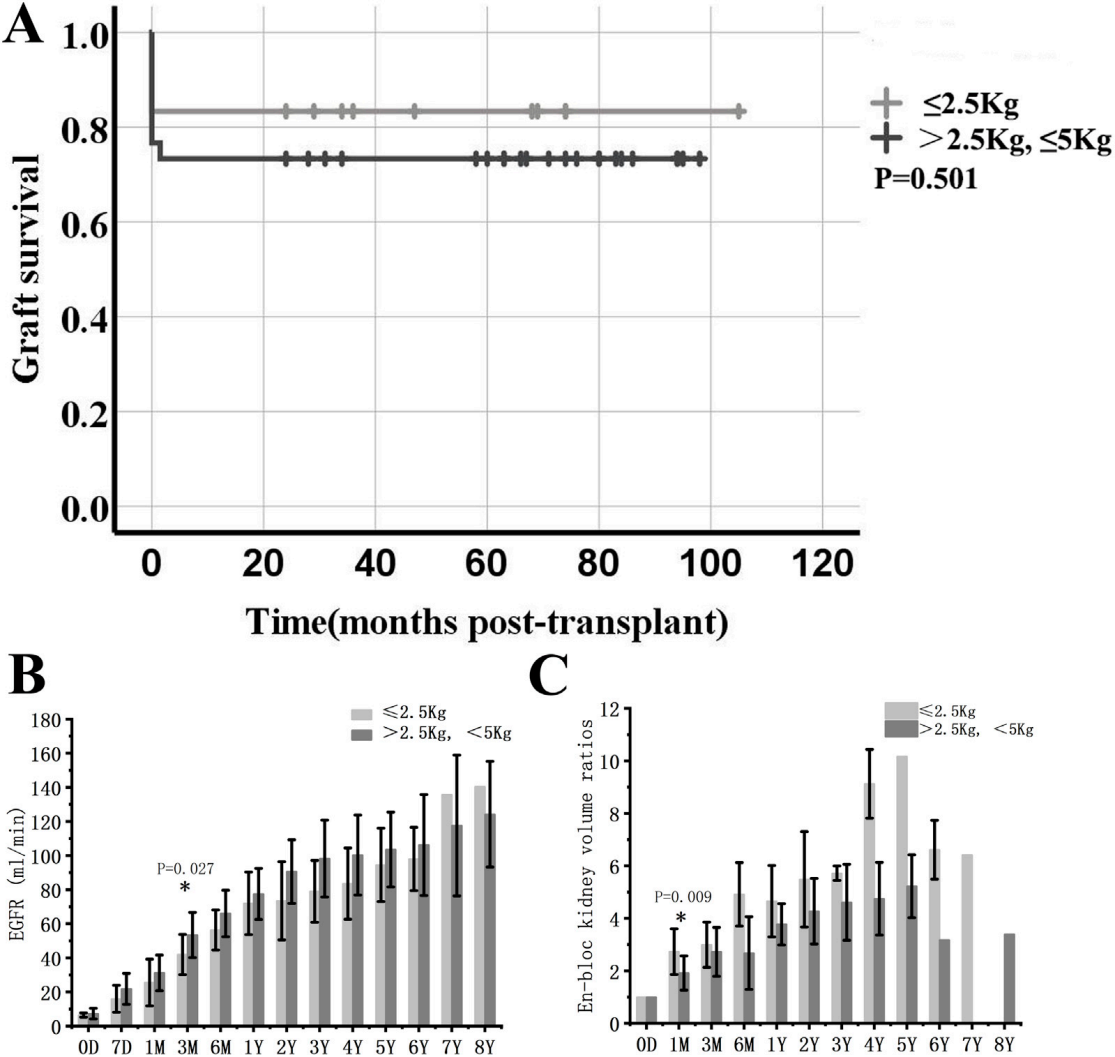
Comparison between *en-bloc* kidney transplant from donors weighing less than 2.5 kg and those weighing between 2.5 and 5.0 kg. **(A)** Graft survival. **(B)** eGFR. **(C)** Volume development of the grafts. * indicates a statistically significant difference between donors weighing less than 2.5 kg and those weighing between 2.5 and 5.0 kg.

There were 12 recipients transplanted from donors weighing less than 2.5 kg. Compared with the other 30 cases in which the donor weight was between 2.5 and 5.0 kg, the general profiles of the two groups were similar except for the significant differences in donor weight and D/R BSA. There were no significant differences in the incidence of DGF, thrombosis, urine leakage, perirenal hematoma, acute rejection, and graft survival between the two groups (Table 3; Figure 5A). EGFR was significantly lower in donors weighing less than 2.5 kg at 3 months, but not at later time points. Moreover, the volume of transplanted kidneys showed a faster increase in the same group in the first month, while the difference gradually decreased with prolonged follow-up (Figures 5B,C).

**TABLE 3:** Profiles of donors and recipients in kidney transplantation from donors weighing less than 2.5 kg and those weighing between 2.5 and 5.0 kg.

	Donor weight less than 2.5 Kg (n = 12)	Donor weight between 2.5 and 5.0 Kg (n = 30)	P-value
Donor age (mean, d)	19.6	33.3	0.154
Donor weight (mean, kg)	1.95	3.62	0.000
Donor gender			0.292
Male recipients	6	21	
Female recipients	6	9	
Recipient age (mean, y)	30.8	26.7	0.195
Recipient weight (mean, kg)	49.9	46.5	0.154
Recipient gender			0.316
Male	3	13	
Female	9	17	
D-R BSA ratio	0.114	0.160	0.000
WIT (mean, min)	10.3	10.5	0.936
CIT (mean, h)	10.9	11.2	0.801
Mean time since first *en-bloc* KTx (mean, d)	1922	1,448	0.071
DGF	5/10	5/22	0.217
Urinary leakage	0/10	5/22	0.155
Perirenal hematoma	1/10	1/22	0.534
Thrombosis	0/12	6/30	0.159
Acute rejection	2/12	2/30	0.585
Graft loss	2/12	8/30	0.696

D/R BSA, donor/recipient body surface area; WIT, warm ischemia time; CIT, cold ischemia time; KTx, kidney transplantation; DGF, delayed graft function.

## Discussion

To our knowledge, the 42 *en-bloc* KTx cases in this retrospective study not only include one with the lowest donor body weight (0.9 kg), but also offer an opportunity to look deep into KTx from the lowest average donor body weight ever reported [6]. With a concerning 23.8% *en-bloc* graft failure rate in donors weighing less than 5 kg, thrombosis and acute rejection are the leading causes of short-term graft loss. However, the remaining 76.2% of cases have satisfactory long-term outcomes. This study suggests that the selection of female recipients and adequate immunosuppressive exposure could potentially further reduce short-term graft loss. DGF does not affect graft survival and long-term graft function in *en-bloc* KTx from low-weight pediatric donors. Lower D/R BSA, which means greater size disparity between donor and recipient, may lead to a higher probability of receiving postoperative dialysis and slower eGFR recovery.

The small kidneys have demonstrated remarkable recovery and growth potential, which is the basis for our clinical application of the proposed procedure [7]. In terms of growth rate, small allografts showed a rapid development during the first 1–3 months, with an approximately 2-fold volume increase followed by a continuous growth trend during the first 1 or even 2 years. Although *en-bloc* KTx from pediatric donors to adult recipients has been performed for over five decades, this procedure remains underutilized globally due to concerns regarding the risks of insufficient nephron mass, renal dysplasia, thrombosis, hyperfiltration injury, and urinary complications [3, 8]. Currently, the reported minimum donor weight is 1.07 kg, and the majority of scholars have reported the outcomes of kidney transplantation using donors weighing between 10 and 20 kg^6^. Although a low body weight donor *en-bloc* renal graft is considered to be one of the expanded criteria donations (ECD), previous studies reported that it has a higher anticipated eGFR than other ECD cases due to a larger reservoir and the fact that it is free of chronic injury [9]. In addition, it may take over a year following KTx to achieve *en-bloc* allograft stability in terms of eGFR, which is significantly longer than with adult grafts.

We prefer adult over pediatric patients as *en-bloc* KTx recipients for the reasons that follow. The first is an ethical consideration. It is more appropriate for the pediatric recipients to receive standard criteria donation instead of ECD. Considering the potential higher risk of short-term graft loss, higher prevalence of DGF and a longer period of eGFR recovery, *en-bloc* KTx from low-weight donors is more acceptable for adult recipients. Second, adult recipients may have more satisfactory long-term clinical outcomes [10, 11]. According to previous reports, with the exception of premature infants, the number of nephrons in full-term infants has reached adult levels, with subsequent growth consisting solely of nephron hypertrophy [12]. Moreover, in this study, even the adult recipients from donors weighing less than 2.5 kg were found to have non-compromised graft function and limited hyperfiltration injury. Thus, our study suggests that the small kidneys from low-weight pediatric donors may have enough adaptability to swiftly meet the needs of the recipients after transplant.

In the analysis of risk factors for short-term graft loss, we found that female recipients exhibited a significantly lower risk compared to male ones. This finding may optimize the inclusion criteria of recipients. While the underlying causes remain unconfirmed, we speculate that this may be attributed to hormonal differences. Previous research has highlighted the role of estrogen as a potent antioxidant within the renal mesangial microenvironment, promoting nitric oxide release through endothelial nitric oxide release. In the context of KTx from low-weight donors, estrogen in female recipients may mitigate donor kidney injury by inhibiting oxidative stress, preserving microvascular integrity, and reducing thrombosis occurrence [13, 14]. In addition, it has been reported that myosteatosis and sarcopenia are associated with an increased risk of mortality in both the pre-transplant waiting group and post-transplantation recipients [15]. Preoperative reduction in muscle mass is linked to a poorer prognosis following kidney transplantation [16]. In our study, the mean body weight of male recipients was 50.1 kg, while the mean body weight of female recipients was 45.9 kg. When compared to the general population, the difference in body weight between male recipients and typical men was more pronounced. This suggests that male recipients may have less muscle mass relative to normal values. Consequently, this factor could potentially contribute to the higher risk of graft loss observed in men compared to women in this study.

Pediatric *en-bloc* KTx poses challenges due to the high incidence of thrombosis in the early postoperative stage. Previous studies have reported thrombosis rates ranging from 2% to 25% within 3 days of surgery, particularly in pediatric donors weighing less than 5 kg [17]. However, the use of anticoagulation therapy during pediatric *en-bloc* KTx still remains controversial [18]. This study reveals that the perioperative administration of LMWH has the risk of perirenal hematoma, and does not contribute to the prevention of postoperative thrombosis, which is consistent with a previous study [19]. This explains our decision to administer LMWH perioperatively in the first cohort of 19 patients, whereas it was not utilized in the subsequent group of 23 patients. A delicate surgical technique is still one of the prerequisites for minimizing *en-bloc* graft loss due to thrombosis. In short, proper aortic cannula for adequate graft flushing during procurement, avoidance of excessive exposure of renal vessels during preparation, and maximization of the venous anastomosis site during implantation are major considerations during the steps [4, 20]. Unlike the adult kidney, it may be much more difficult to detect the torsion of the small grafts during the procedure. Adequate vascular surrounding tissue, keeping the bilateral ureters attached to the donor bladder before ureteral reconstruction may be helpful for prevention. Moreover, before closing the incision, it is vital to optimize the relative positions of the two grafts and permit further growth of the grafts, vessels and ureters.

In addition, pediatric donor kidneys have a much higher risk of graft loss due to acute rejection compared to adult donor kidneys [21]. As we have shown in this study, all three recipients with acute rejection experienced graft loss shortly after the procedure. The disparity may be due to the fragility of the small grafts and the fluctuation in Tac blood levels. However, it remains unknown whether the fast-growing grafts play an additional role in the fluctuation. Considering the devastating outcomes, adequate immunosuppressive exposure is vital. Thus, we suggest daily monitoring of Tac levels to assure adequate Tac exposure in the short period after KTx. Additionally, we recommend a more intensive immunosuppressive regimen as a viable strategy.

DGF may be a risk factor for long-term renal allograft failure in adult KTx outcomes. However, it may not be true in the setting of pediatric *en-bloc* KTx [22]. We found that there was no significant difference in eGFR level between the DGF group and the non-DGF group for 1 month post-KTx, and the long-term graft survival rate was also similar. However, the D/R BSA ratio may serve as an effective indicator for predicting short-term postoperative renal function recovery [23]. In this study, the recipients with higher D/R BSA were less likely to need dialysis after KTx. This suggests that recipients with lower body weight may have a smoother recovery. Significantly faster graft volume growth in the DGF group in the first month could be due to a stronger driving force in the”, which are adapted to much larger recipients.

Pediatric *en-bloc* renal allografts are associated with a potentially higher incidence of postoperative urinary complications [24]. However, these complications can generally be successfully treated non-surgically, and there is no evidence of a negative impact on allograft survival rate [5]. Our findings are consistent with this conclusion. Except for one case of postoperative long-segment necrosis resulting from intraoperative injury, the other four cases of urinary leakage resolved by prolonging the indwelling time of the urinary catheter. The following strategies may be advantageous in minimizing ureteral complications: (1) Preserving adequate ureteric surrounding tissue during organ procurement to ensure a rich blood supply. (2) Shortening the internal ureter before ureteroneocystostomy. (3) Avoiding forceful stent placement to minimize the possibility of mechanical damage to the ureter. (4) Shortening the graft CIT [25].

Previous research found significantly higher levels of proteinuria in pediatric *en-bloc* KTx patients compared to adult KTx patients at 6 and 12 months [26]. To mitigate hyperfiltration injury, another study showed benefits of maintaining systolic blood pressure below 130 mmHg^4^. We did not detect significant glomerulosclerosis in the pathological findings of the five recipients who underwent indicative biopsy approximately 2 years after transplantation (Supplementary Material S5). Consequently, we conclude that hyperfiltration injury in recipients is tolerable, provided that blood pressure is rigorously maintained below 140 mmHg following kidney transplantation. Furthermore, none of the proteinuric patients developed edema or hypoalbuminemia, which is consistent with common findings in maladaptive focal segmental glomerulosclerosis [27].

In this study, donor body weight was found not to represent a risk factor for graft loss. Pediatric donors weighing less than 2.5 kg had comparable graft survival and graft function outcomes to the donors weighing between 2.5 and 5.0 kg. Although KTx from these donors poses challenges during the surgical procedure and has a slower eGFR increase, long-term outcomes are not compromised. Given these findings, we propose that the utilization of lower body weight (<2.5 kg) kidneys should be determined based on the related experience of individual transplant teams, and the preferences of the recipients. While these grafts may present challenges, they should not be a contraindication to KTx.

Our study has several limitations, including its retrospective nature and the limited number of cases and follow-up period. In our study, the mean WIT was 10 min, and the mean CIT reached 11 h. Therefore, the use of the University of Wisconsin solution and hypothermic machine perfusion may potentially enhance postoperative outcomes. To address these limitations, a comprehensive follow-up study will be conducted over an extended period. Furthermore, it would be more interesting if the follow-up also included pathological findings.

In conclusion, although instances of graft failure and severe complications occur primarily in the initial stages, KTx from extremely low-weight donors can still expand the donor pool and have promising long-term graft function. A body weight of less than 2.5 kg should not be an absolute contraindication for kidney donation. The clinical recommendations offered in this study could further optimize the clinical outcomes of this procedure. Furthermore, considering the unique physiology, pathology and immunology in the very young and low-weight pediatric donors, this transplant environment may also offer an opportunity to study kidney development and other related issues.

## Data Availability

The raw data supporting the conclusions of this article will be made available by the authors, without undue reservation.
