# Supplementary material for: En-Bloc Kidney Transplantation From Extremely Low-Weight (0.9–5.0 kg) Pediatric Donors: A Decade of Single-Center Experience
Source: Transpl Int. 2025 May 20;38:14451. doi: 10.3389/ti.2025.14451 (PMC12131009; doi:10.3389/ti.2025.14451)
Supplement: Supplementary file 1 [file DataSheet2.PDF]

Supplementary file 2 Risk factors for graft loss after en-bloc kidney transplantation

	Graft survival (n=32)	Graft loss (n=10)	P-Value	95%CI
Donor gender			0.417	0.468 (0.074-2.936)
Male	20	7		
Female	12	3		
Recipient age (mean, y)	27.0	30.8	0.252	0.942 (0.850-1.043)
Recipient gender			0.036	0.161 (0.029-0.884)
Male	9	7		
Female	23	3		
D-R BSA ratio	0.147	0.146	0.984	0.714 (0.000-7.94E+13)
CIT (mean, h)	11.0	11.5	0.435	0.905 (0.704-1.163)
Mean time since the first en-bloc KTx (mean, d)	1673	1299	0.215	1.001 (1.000-1.002)

D/R BSA, donor/recipient body surface area; CIT, cold ischemia time; KTx, kidney transplantation
