# Supplementary material for: En-Bloc Kidney Transplantation From Extremely Low-Weight (0.9–5.0 kg) Pediatric Donors: A Decade of Single-Center Experience
Source: Transpl Int. 2025 May 20;38:14451. doi: 10.3389/ti.2025.14451 (PMC12131009; doi:10.3389/ti.2025.14451)
Supplement: Supplementary file 2 [file DataSheet4.PDF]

Supplementary file 4 Profiles of donors and recipients in DGF and non-DGF group

	DGF (n=10)	Non-DGF (n=22)	P-Value
Donor age (mean, d)	22.7	28.9	0.567
Donor weight (mean, kg)	2.7	3.3	0.133
Donor gender			0.248
Male	8	12	
Female	2	10	
Recipient age (mean, y)	28.6	26.2	0.473
Recipient weight (mean, kg)	49.8	45.3	0.088
Recipient gender			0.681
Male	2	7	
Female	8	15	
D-R BSA ratio	0.131	0.154	0.050
Mean time since the first en-bloc KTx (mean, d)	1803	1613	0.519
WIT (mean, min)	10.0	10.5	0.800
CIT (mean, h)	10.1	11.1	0.420
Urine leakage	2/10	3/22	0.637
Perirenal hematoma	1/10	1/22	0.534

D/R BSA, donor/recipient body surface area; KTx, kidney transplantation; WIT, warm ischemia time; CIT, cold ischemia time
