# Supplementary material for: En-Bloc Kidney Transplantation From Extremely Low-Weight (0.9–5.0 kg) Pediatric Donors: A Decade of Single-Center Experience
Source: Transpl Int. 2025 May 20;38:14451. doi: 10.3389/ti.2025.14451 (PMC12131009; doi:10.3389/ti.2025.14451)
Supplement: Supplementary file 3 [file DataSheet6.PDF]

Capsule Sentence Summary (40 words):

Forty-two en-bloc kidney transplants from extremely low-weight pediatric donors (0.9-5.0kg) achieve 76.2% long-term graft survival. The grafts undergo at least one year of growth and renal function recovery in the adult recipients, expanding donor pool despite early risks.

Graphical Abstract

En-Bloc Kidney Transplantation from Extremely Low-Weight Pediatric Donors (0.9-5.0kg): A Decade of Single-Center Experience

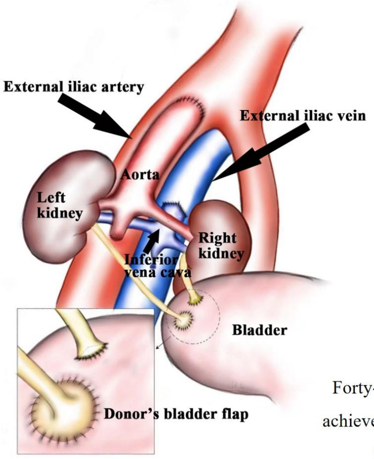

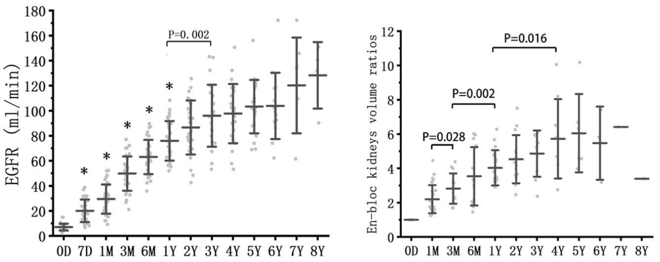

Forty-two en-bloc kidney transplants from extremely low-weight pediatric donors (0.9-5.0kg) achieve 76.2% long-term graft survival. The grafts undergo at least one year of growth and renal function recovery in the adult recipients, expanding donor pool despite early risks.
