# Supplementary material for: En-Bloc Kidney Transplantation From Extremely Low-Weight (0.9–5.0 kg) Pediatric Donors: A Decade of Single-Center Experience
Source: Transpl Int. 2025 May 20;38:14451. doi: 10.3389/ti.2025.14451 (PMC12131009; doi:10.3389/ti.2025.14451)
Supplement: Supplementary file 4 [file DataSheet3.PDF]

Supplementary file 3 Risk factors for thrombosis after en-bloc kidney transplantation

	Non-thrombosis (n = 36)	Thrombosis (n = 6)	P-Value	95%CI
Donor gender			0.345	3.555 (0.255-49.537)
Male	22	5		
Female	14	1		
Recipient age (mean, y)	27.8	28.3	0.566	0.951 (0.800-1.130)
Recipient gender			0.187	4.771 (0.468-48.645)
Male	12	4		
Female	24	2		
D-R BSA ratio	0.144	0.159	0.343	0.000 (0.000-7.058E+10)
CIT (mean, h)	10.9	12.2	0.205	0.805 (0.576-1.125)
LMWH application	14	5	0.961	1.095 (0.028-42.257)
Mean time since the first en-bloc KTx (mean, d)	1685	975	0.240	1.002 (0.999-1.005)

D/R BSA, donor/recipient body surface area; CIT, cold ischemia time; LMWH, low molecular weight heparin; KTx, kidney transplantation
