# Supplementary material for: En-Bloc Kidney Transplantation From Extremely Low-Weight (0.9–5.0 kg) Pediatric Donors: A Decade of Single-Center Experience
Source: Transpl Int. 2025 May 20;38:14451. doi: 10.3389/ti.2025.14451 (PMC12131009; doi:10.3389/ti.2025.14451)
Supplement: Supplementary file 5 [file DataSheet1.pdf]

Supplementary Material S1

Certificate of approval

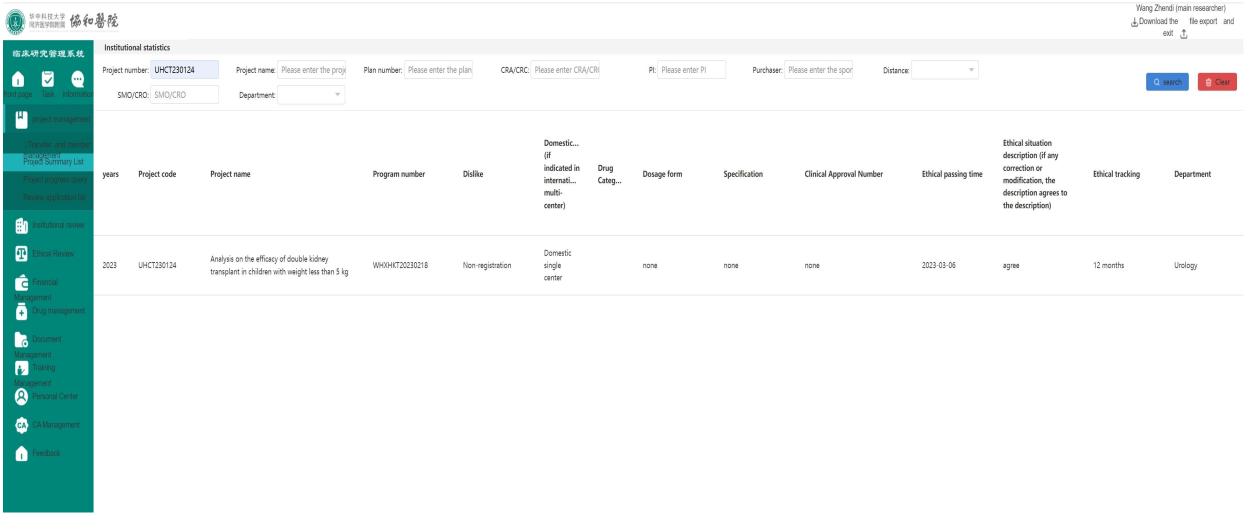

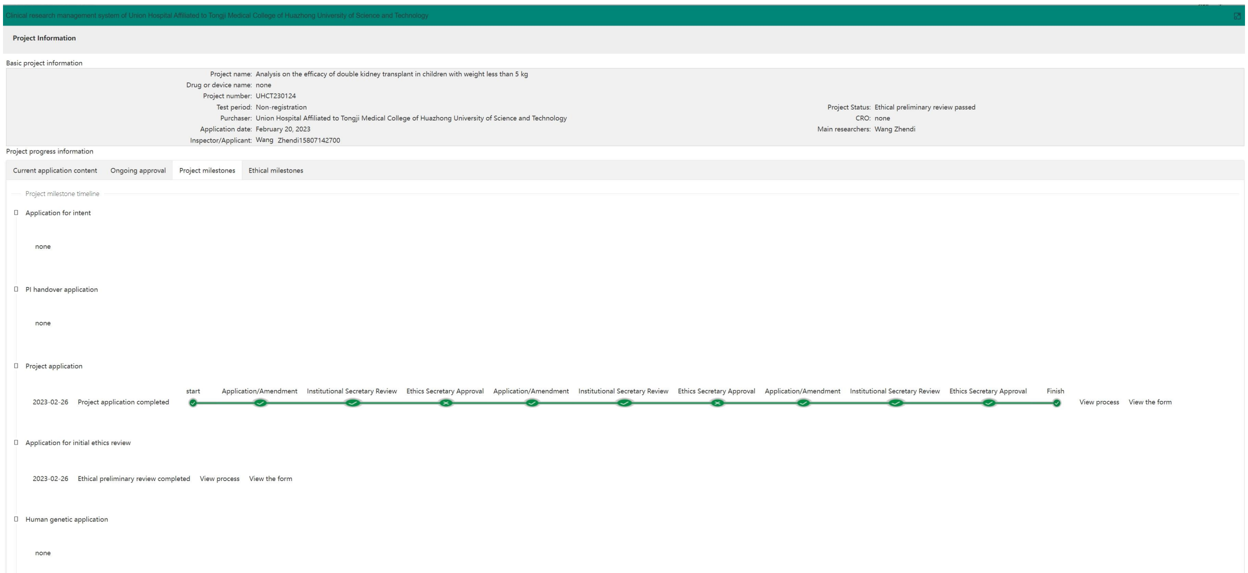

Project Proposal in English

Hua Zhong University of Science and Technology Tongji Medical College Affiliated Union Hospital

Clinical research project protocol

project name	Analysis of the efficacy of en-bloc kidney transplantation in adults from donors weighing less than 5 kg
Programme number	WHXHKT20230218
version number	V1.0
Version date	20230218
Lead researcher	Wang Zhendi
Professional departments	urinary surgery
Estimated start	2023.4-2023. 10

and end dates

Signature page of the programme

Project name: Analysis of the efficacy of en-bloc kidney transplantation in adults from donors weighing less than 5 kg

Version number/version date: V1.0/20230218

I have read and understood this research protocol, confirming that it includes all necessary elements for the implementation of the study and clearly defining the responsibilities of researchers related to this protocol. As the principal investigator, I will provide copies of this protocol and all relevant materials to all participants in this study. I will discuss these materials with them to ensure they fully understand how the protocol will be implemented. I agree to strictly adhere to current applicable laws and regulations, the Helsinki Declaration, Good Clinical Practice, and this research protocol in fulfilling my duties.

Signature (print) of the principal investigator: Wang Zhendi

Lead investigator signature/ date:

clinical research unit:

catalogue

Signature page of the programme 3

scenario summary 7

Information on principal investigators 8

1. foreword 11

1.1 Research background 11

1.1.1 Basic overview 11

1.2 Types of research 11

1.3 Research basis 11

1.4 Risk/benefit assessment 12

1.4.1 Known potential risks 12

1.4.2 Probability of injury 12

1.4.3 The degree of injury 12

1.4.4 Known potential benefits 13

1.4.5 Potential risk/benefit assessment 13

1.4.6 Discussion 13

2. Study objectives/endpoints 14

3. research design 14

3.1 Overall design 14

3.2 Sample size 14

4. study population 15

4.1 Diagnostic criteria 28

4.2 Selection criteria 15

4.3 Exclusion criteria 15

5. appraise 15

5.1 Primary and secondary endpoints/outcomes evaluation 15

5.2 Safety evaluation 15

6. Adverse events and serious adverse events 16

7. Statistical analysis and statistical methods 16

8. Medical care and protection for subjects 16

8.1 Assessment of risks to subjects in the study and risk management measures and contingency plans 16

9. Supporting documents and notes 17

9.1 Privacy protection 17

9.2 Collection and use of specimens and data 17

9.3 Quality control and quality assurance 17

9.4 Data processing and record keeping 17

9.4.1 Data collection and management 17

9.4.2 Study data retention 18

9.5 Conflict of interest statement 18

10. reference documentation 18

scenario summary	
**Study name**	Analysis of the efficacy of en-bloc kidney transplantation in adults from donors weighing less than 5 kg
**Research overview**	Because of the great difficulty of surgery, en-bloc kidney transplantation from donors weighing less than 5 kg has not been widely used in clinical practice. In order to expand the source of kidney supply, this study retrospectively analyzed the postoperative efficacy of 42 cases of en-bloc kidney transplantation from donors weighing less than 5 kg, analyzed the postoperative efficacy, and summarized the surgical experience.
**purpose of research**	This paper summarizes the clinical application data and experience of single-center en-bloc kidney transplantation from donors less than 5Kg, and discusses the feasibility and efficacy of extremely low weight kidney transplantation.
**research design**	retrospective study
**Total number of patients enrolled**	42
**Number of research groups**<br>**Number of control groups**	42
**diagnose**	toxuria
**Selection criteria**	Pediatric donors with a body weight of less than 5 kg / uremic patients
**Exclusion criteria**	A recipient who is not a candidate for transplant surgery
**Study interventions**	not have

**evaluation criterion:**<br>**Primary endpoint:**<br>**Secondary endpoint:**<br>**safety evaluation:**	Primary endpoint: short-and long-term graft survival<br>Secondary endpoints: postoperative complication rates, changes in graft kidney volume, and estimated glomerular filtration rate levels<br>Safety evaluation: survival rate of transplant recipients and incidence of postoperative complications
**statistical method:**<br>**sample capacity:**<br>**analytic set:**<br>**Main efficacy endpoint:**<br>**Safety endpoint:**	Sample size: 42 cases of nephrectomy were performed on all pediatric donors weighing less than 5 kg in this center;<br>Method: The measurement data of both kidneys from pediatric donors were expressed as mean ± standard deviation, the count data were expressed as frequency, the measurement data were applied t test, and the count data were applied to the square root analysis<br>Main efficacy endpoint: short-and long-term graft survival<br>Safety endpoint: None
**Study duration**	2023.4-2023.10
**Time of participant participation**	2023.4-2023.10
**Research unit/ site**	Domestic single center: Union Hospital affiliated to Tongji Medical College of Huazhong University of Science and Technology

Information on principal investigators
Name, qualification and contact information of the main researcher<br>Main investigator: Wang Zhendi; Doctor, associate professor, 15807142700

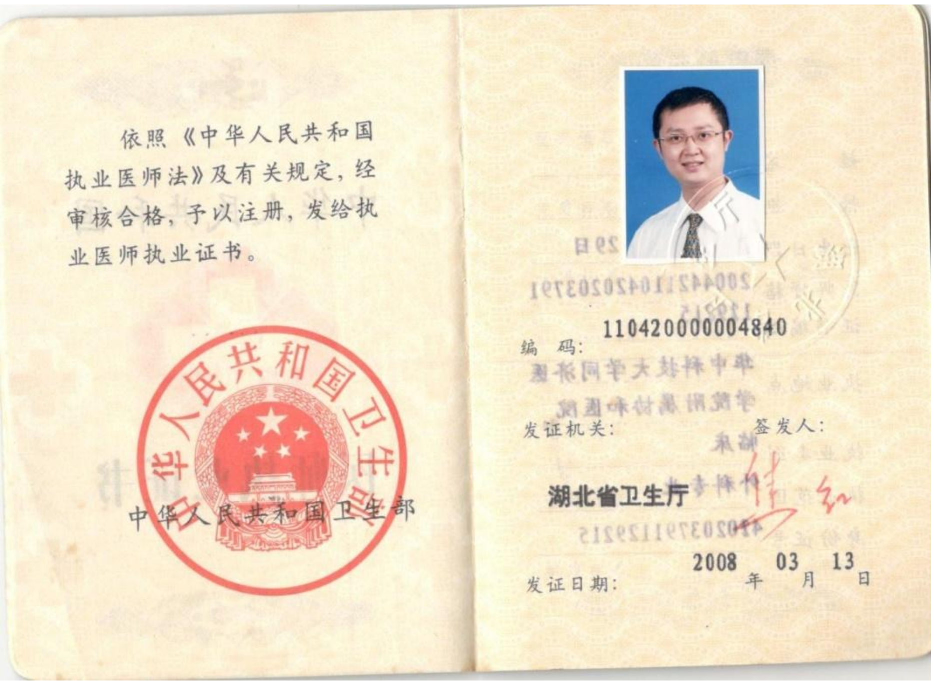

依照《中华人民共和国执业医师法》及有关规定，经审核合格，予以注册，发给执业医师执业证书。

中华人民共和国卫生部

编　码：110420000004840

发证机关：湖北省卫生厅

签发人：

发证日期：2008 年 03 月 13 日

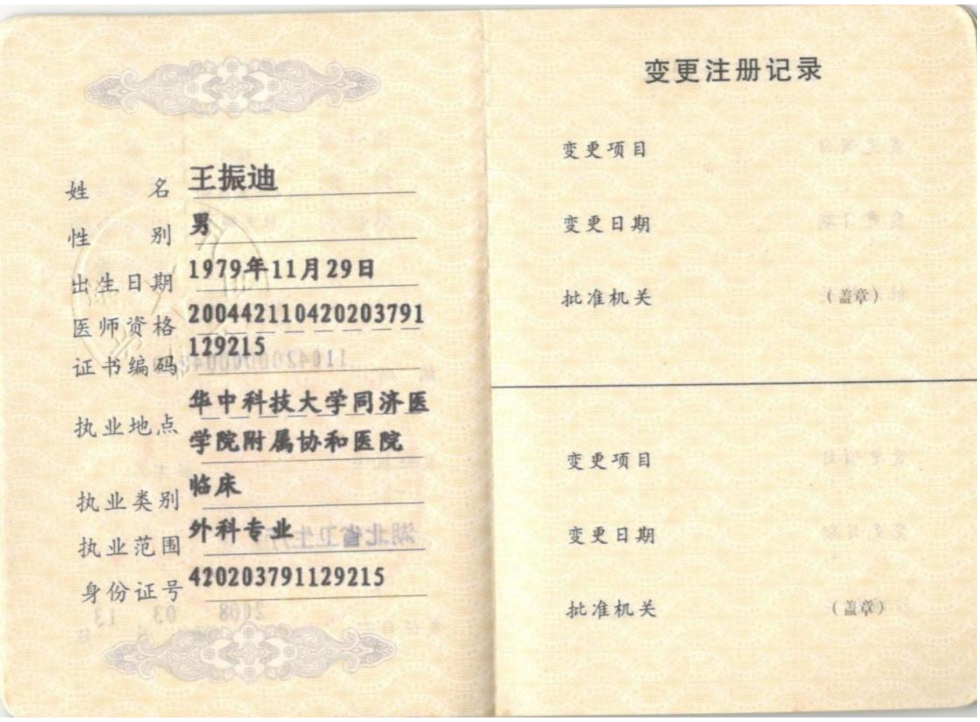

姓　　名　王振迪

性　　别　男

出生日期　1979年11月29日

医师资格证书编码　200442110420203791129215

执业地点　华中科技大学同济医学院附属协和医院

执业类别　临床

执业范围　外科专业

身份证号　420203791129215

变更注册记录

变更项目

变更日期

批准机关　（盖章）

变更项目

变更日期

批准机关　（盖章）

国家食品药品监督管理局培训中心

培训证书

王振迪 同志于 2009 年 7 月 4 日至

2009 年 7 月 7 日参加 药物临床试验

质量管理规范（GCP）培训班 学习

完成培训计划全部课程。

特发此证

学时：32

学分：10

2009 年 7 月 [illegible] 日

国家食品药品监督管理局培训中心 培训专用章

证书编号：200910623

1. foreword

1.1 research background

1.1.1 Basic overview

In order to expand the pool of transplanted kidneys, special kidney supply has been gradually applied in clinical practice and discussed its efficacy. Among them, pediatric bilateral kidney transplantation has been developed for more than 50 years, and has continuously broken the limitation of body weight and shown promising efficacy.[1]In China, the infant mortality rate is about 1.393%. Considering the huge population base, if such successful kidney supply is applied to clinical practice, it will effectively alleviate the shortage of kidney supply.[2-3]However, worldwide, due to the high technical requirements, more perioperative complications and long-term prognosis concerns, most transplant centers are still cautious about the en-bloc kidney transplantation of low weight pediatric donors, among which, the en-bloc kidney transplantation of pediatric donors weighing less than 5Kg is rarely used.[4-5]

Wijetunga et al[6]A comparative analysis was conducted on the outcomes of en-bloc kidney transplants in 15 donors weighing less than 5 kg and 15 donors weighing between 5 kg and 20 kg. The one-year survival rates for the two groups were 86.7% and 93.3%, respectively (P = 0.85). Although donors weighing less than 5 kg have a longer recovery period for kidney function and a higher risk of early graft loss, they remain a promising source of donor kidneys.

1.2 Study type

observational study

Retrospective analysis of the efficacy of transplants in adult uremia patients with a body weight of less than 5Kg.

1.3 Research basis

Existing literature has reported that pediatric en-bloc kidney transplantation is

superior to living donor kidney transplantation in terms of long-term postoperative outcomes. Due to the complex operational requirements, there is currently no systematic experience or efficacy to draw upon. This study summarizes the outcomes of en-bloc kidney transplantation using donors weighing less than 5 kg, analyzes risk factors for postoperative complications, explores the feasibility of kidney transplantation from pediatric donors, and aims to promote its application.

1.4 Risk/benefit assessment

1.4.1 Known potential risks

This study is an observational study. All the operations and treatments have been completed, and only medical records and follow-up information are collected. There are no intervention measures for patients and no operational risks.

The subjects are at risk of privacy leakage. In the process of collecting medical records and follow-up information, the basic information of the relevant donor and recipient as well as the surgical data may be leaked due to the negligence of the collector, the theft or theft of the stored data.

For the already implemented pediatric en-bloc kidney transplantation, there are reports that the risk of postoperative failure of the transplanted kidney, thrombosis, delayed recovery of the transplanted kidney function, acute rejection reaction, urine leakage and other complications may increase. This type of organ belongs to the clinically approved source of donor kidney.

1.4.2 The probability of injury

The study will not cause harm to patients and there is a rare risk of disclosure of subject information.

1.4.3 The extent of the harm

The observed patients will not suffer from operational injuries, and if information leakage occurs, there is a possibility of mild physical and mental harm to the subjects, but no fatal adverse events.

1.4.4 Known potential benefits

The study retrospectively analyzed the short-term and long-term outcomes of surgical patients, aiming to benefit subsequent treatment through improved disease management. The primary investigators were the direct surgeons who performed the surgeries, and patients still required follow-up care from these surgeons. With repeated familiarity with the patients medical history, relevant diagnostic and therapeutic measures would be more precise;

This study summarizes the prognosis and experience of related surgery, which is beneficial to the follow-up implementation of this type of surgery, that is, through the summary and analysis of existing data, the risk factors of this surgery can be judged to reduce the possible exposure risk of patients in subsequent treatment;

As for the surgery itself, a large number of studies have shown that kidney transplantation can significantly improve the survival rate of uremic patients compared with regular dialysis, and the implementation of such surgery expands the source of donor kidney, which is beneficial to the patients who undergo the operation; at the same time, pediatric donors with long-term survival of kidney recipients may have better survival time of transplanted kidneys than those with relatives kidney transplantation recipients.

1.4.5 Potential risk/benefit assessment

The observational study requires regular review of the reliability and security of data collection, regular check of the reliability of data storage, and assessment of information leakage risk. After data analysis and sorting, subjects can benefit from diagnosis and treatment of their conditions.

1.4.6 discussion

The application of pediatric kidney donation as an effective method to expand the source of kidneys has gained widespread recognition. For pediatric donors weighing less than 5Kg, due to the high technical difficulty, there are few reports. From known experience, pediatric donors weighing less than 5Kg may increase the risk of postoperative complications; however, compared to dialysis, it can significantly improve survival rates for uremic patients. In the context of a shortage of

kidneys, this is an effective way to expand the source of kidneys.

This observational study only collects data from the medical record system and the national follow-up system, and will not pose a risk to patients. In addition, through systematic review of patients, it may be beneficial for subsequent treatment of the disease, and the conclusions of the study are beneficial for the continued development of such surgery.

2. Study objectives/endpoints

Main objective: To summarize and analyze the survival rate of short and long term grafts, and to evaluate the feasibility of en-bloc kidney transplantation from pediatric donors weighing less than 5Kg.

Secondary purpose: To understand the safety and effectiveness of this type of donor kidney by summarizing and analyzing the incidence of post-transplantation complications and the growth and development of transplanted kidneys.

3. research design

3.1 system design

This study is an observational case review study that retrospectively analyzes the relevant clinical data of previous operations of this type and summarizes experience

The study involved four researchers responsible for data collection, which came from two sources: the inpatient medical record system and the national kidney transplant patient follow-up system. The data collection criteria are detailed in the case information form, and after collection, they were organized and archived by designated personnel for analysis. All data collection and organization were kept confidential from external researchers.

3.2 sample capacity

A total of 42 cases of transplantation data have been collected since the operation was carried out.

4. study population

4.1 Donor and recipient

Donor: Pediatric donors with a body weight less than 5Kg. The donor selection criteria are in line with the organ donation standards for Chinese citizens after death.

Recipient: uremic patient

4.2 Selection criteria

Patients with uremia due to primary kidney disease, genetic diseases, metabolic diseases, systemic diseases and obstruction who undergo surgery after being informed of the type of donor and surgical risks.

4.3 Exclusion criteria

In the screening of pediatric donor kidney recipients, we follow the following exclusion criteria in addition to applying conventional renal transplant exclusion criteria: (1) refractory hypertension and long-standing uncontrolled diabetes, coronary heart disease history or peripheral vascular disease history, hypercoagulable state, urinary abnormalities; (2) PRA> 10%, positive lymphotoxicity test, second or multiple transplants, lupus erythematosus; (3) poor compliance in the past.

5. Appraise

5.1 Primary and secondary endpoints/outcomes

Main endpoint: short and long term graft survival, renal transplant recipient survival.

Secondary endpoint: postoperative complication rate (delayed graft function, rejection reaction, bleeding, urinary tract complications, etc.), growth of transplanted kidney and renal function recovery.

5.2 Safety evaluation

The survival rates of kidney transplant recipients were compared with those of published uremic patients.

6. Adverse events and serious adverse events

The study was an observational study, and adverse events included data collection errors, data leakage, and data analysis errors.

For the operation itself, adverse events: postoperative complications such as delayed graft function, rejection reaction, bleeding, urinary leakage and other postoperative complications occurred

Serious adverse events: failure of transplanted kidney, death of transplant recipient

7. Statistical analysis and statistical methods

All statistical analyses were conducted using SPSS 22 and Windows Origin 9.0 versions. All quantitative data were expressed as mean and standard deviation. The Kaplan-Meier survival curve was used to evaluate differences in graft survival rates. Quantitative data were analyzed using the chi-square test, and t-tests were used for comparing two independent samples. Results were considered statistically significant if $P < 0.05$.

8. Medical care and protection for subjects

8.1 Risk assessment of subjects in the study and risk management measures and plans

Data collection errors and data analysis errors

Evaluation and disposal measures: A total of 4 people collected data, and each piece of data was checked at least twice after collection. After the data collection was completed, the dispersion of the data was evaluated uniformly. When obvious abnormal data was found, the original data was traced back and modified at the same time.

Data leakage

Evaluation and disposal measures: All data collected are uniformly saved on one computer and an electronic backup. The password is set for the data folder, which is

only available for the researcher to use.

9. Supporting documents and notes

9.1 privacy protection

All data in this study are available only to the applicants and participants, and the information protection of donors and recipients has been clearly informed to the participants.

9.2 Collection and use of specimens and data

There is no third party use of data in this study and all data are for the use of this study only.

9.3 Quality control and quality assurance

1. This type of kidney supply has been approved by Chinas organ acquisition organization and is one of the legal sources of kidney supply;

2. The operation method has been approved by the application of new technology in our hospital in the early stage;

3. Approved by the organ transplantation ethics committee of our hospital;

4. Strict data collection standards, four people collect data and compare each other to get the final retrospective analysis results;

5. Follow-up data after transplantation should be managed by specialists, and the recovery of patients creatinine should be recorded regularly. At the same time, the data should be consistent with the national system to ensure the authenticity and reliability of the data.

9.4 Data processing and record keeping

9.4.1 Data collection and management

Organize the patients past medical records, collect relevant data for the proposed study, and gather prognosis information; long-term follow-up data is queried through the China Organ Transplant Follow-up Registration System. For each patient who has undergone kidney transplantation, follow-up data must be uploaded to the system by designated personnel. In this study, a total of three individuals were involved in data

collection and organization, and they mutually verified whether there were any errors in data entry.

9.4.2 Study data retention

All medical records have been saved in the computer in the form of pictures, and patient follow-up data have been stored in the Chinese Organ Transplant Follow-up Registration System.

9.5 Conflict of interest statement

A conflict of interest statement has been signed, see annex.

10. reference documentation

[1] Pelletier S J, Guidinger M K, Merion R M, Pelletier S J, Guidinger M K, Merion R M, et al. Recovery and utilization of deceased donor kidneys from small pediatric donors[J]. Am J Transplant, 2006,6(7):1646-1652.

[2] Meakins J L, Smith E J, Alexander J W. En bloc transplantation of both kidneys from pediatric donors into adult patients[J]. Surgery, 1972,71(1):72-75.

[3] Dai H, Peng L, Peng F, Dai H, Peng L, Peng F, et al. A novel technique for en bloc kidney transplantation from infant donors with extremely low body weight by using the distal abdominal aorta as an outflow tract[J]. Am J Transplant, 2018,18(9):2200-2207.

[4] Wijetunga I, Pandanaboyana S, Farid S G, Wijetunga I, Pandanaboyana S, Farid S G, et al. Neonatal kidney donation and transplantation: a realistic strategy for the treatment of end-stage renal disease[J]. Arch Dis Child Fetal Neonatal Ed, 2014,99(6):F518-F519.

[5] Mohanka R, Basu A, Shapiro R, Mohanka R, Basu A, Shapiro R, et al. Single versus en bloc kidney transplantation from pediatric donors less than or equal to 15 kg[J]. Transplantation, 2008,86(2):264-268.

[6] Wijetunga I, Ecuyer C, Martinez-Lopez S, et al. Renal transplant from infant and neonatal donors is a feasible option for the treatment of end-stage renal disease but is associated with increased early graft loss[J]. Am J Transplant, 018,18(11):2679-2688.

Project Proposal in Chinese

华中科技大学同济医学院附属协和医院

临床研究项目方案

项目名称	体重小于 5 公斤小儿双供肾移植成人疗效分析
方案编号	WHXHKT20230218
版本号	V1.0
版本日期	20230218
主要研究者	王振迪
专业科室	泌尿外科
预计起止年月	2023.4-2023.10

方案签字页

项目名称：体重小于 5 公斤小儿双供肾移植成人疗效分析

版本号/版本日期：V1.0/20230218

我已阅读、理解本研究方案，确认方案中包括研究实施的必要内容，明确与本研究方案有关的研究者职责。作为主要研究者我将向所有参加本研究的研究人员提供此方案的复印件及所有相关资料。我将与他们讨论这些资料，以保证他们完全了解本研究方案如何实施。我同意并将严格按照当前适用的法律法规、赫尔辛基宣言、药物临床试验质量管理规范以及本研究方案履行相关的职责。

主要研究者签名（打印）：王振迪

主要研究者签名/日期：

临床研究单位：

目录

方案签字页 3

方案摘要 7

主要研究者资料 8

1. 引言 11

1.1 研究背景 11

1.1.1 基本概况 11

1.2 研究类型 11

1.3 研究依据 11

1.4 风险/获益评估 12

1.4.1 已知的潜在风险 12

1.4.2 伤害的概率 12

1.4.3 伤害的程度 12

1.4.4 已知的潜在获益 13

1.4.5 潜在风险/获益评估 13

1.4.6 讨论 13

2. 研究目的/终点 14

3. 研究设计 14

3.1 总体设计 14

3.2 样本量 14

4. 研究人群 15

4.1 诊断标准 28

4.2 入选标准 15

4.3 排除标准 15

5. 评价 15

5.1 主要及次要终点/结局评价 15

5.2 安全性评价 15

6. 不良事件与严重不良事件 16

7. 统计分析与统计方法 16

8. 受试者的医疗与保护 16

8.1 受试者在研究中风险的评估以及风险处置措施、预案 16

9. 支持性文件与注意事项 17

9.1 隐私保护 17

9.2 标本、资料的收集与使用 17

9.3 质量控制与质量保证 17

9.4 数据处理与记录保存 17

9.4.1 数据收集与管理 17

9.4.2 研究数据保留 18

9.5 利益冲突声明 18

10. 参考文献 18

方案摘要	
研究名称	体重小于5公斤小儿双供肾移植成人疗效分析
研究简介	体重小于5公斤小儿双供肾因手术难度大尚未广泛应用于临床，为扩大供肾来源，本研究回顾性分析42例已完成的体重小于5公斤小儿双供肾移植成人术后疗效，分析术后疗效，总结手术经验。
研究目的	总结单中心小于 5Kg 小儿双供肾移植临床应用数据和经验，探讨极低体重供肾临床应用的可行性及疗效。
研究设计	回顾性研究
入组患者总数	42
研究组数量<br>对照组数量	42
诊断	尿毒症
入选标准	体重小于 5 公斤新生儿供者/尿毒症患者
排除标准	不符合移植手术的受者
研究干预	无
评价标准：<br>主要终点：<br>次要终点：<br>安全性评价：	主要终点：近远期移植物生存率<br>次要终点：术后并发症发生率、移植肾体积变化、估算肾小球滤过率水平<br>安全性评价：移植受者生存率、术后并发症发生率
统计方法：<br>样本量：<br>分析集：<br>主要疗效终点：<br>安全性终点：	样本量：统计本中心所有体重小于 5 公斤新生儿供肾手术共计 42 例；<br>方法：小儿双肾计量资料采用均数±标准差，计数资料以频数表示，计量资料应用 t 检验，计数资料应用卡方分析<br>主要疗效终点：近远期移植物生存率<br>安全性终点：无
研究持续时间	2023.4-2023.10

受试者参加时间	2023.4-2023.10
研究单位/地点	国内单中心：华中科技大学同济医学院附属协和医院

主要研究者资料

主要研究者姓名、资质、联系方式

主要研究者：王振迪；博士、副教授， 15807142700

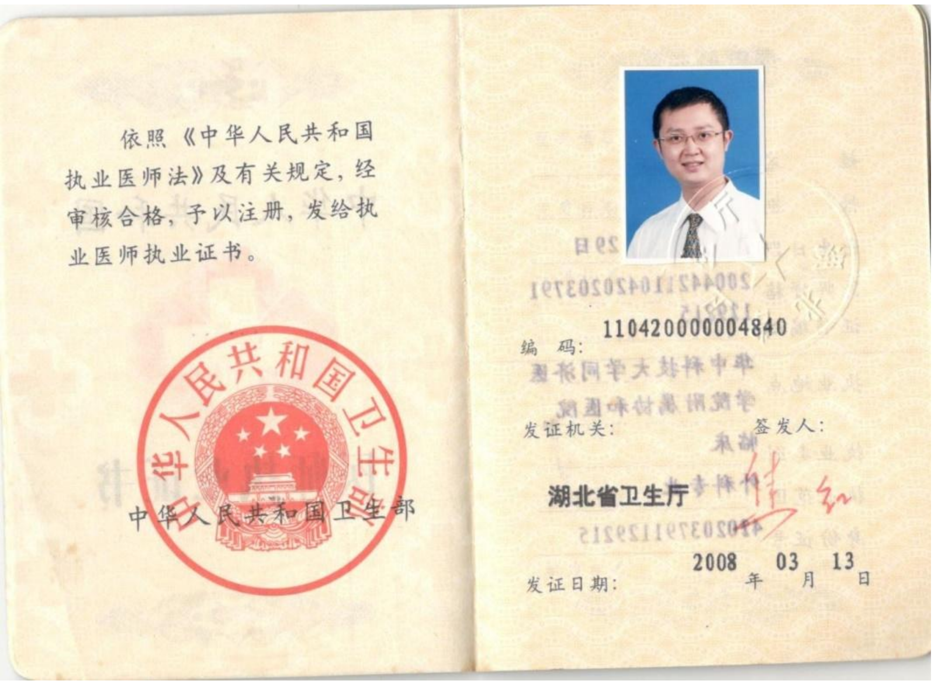

依照《中华人民共和国执业医师法》及有关规定，经审核合格，予以注册，发给执业医师执业证书。

中华人民共和国卫生部

编 码: 110420000004840

发证机关: 湖北省卫生厅

签发人:

发证日期: 2008 年 03 月 13 日

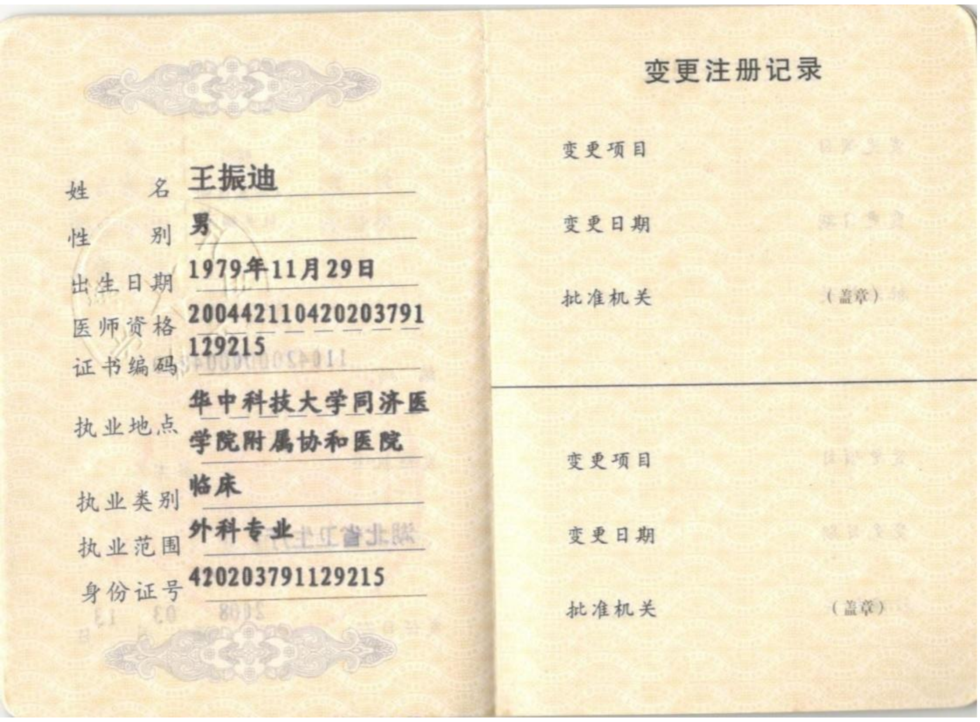

姓 名 王振迪

性 别 男

出生日期 1979年11月29日

医师资格证书编码 200442110420203791129215

执业地点 华中科技大学同济医学院附属协和医院

执业类别 临床

执业范围 外科专业

身份证号 420203791129215

变更注册记录

变更项目

变更日期

批准机关 （盖章）

变更项目

变更日期

批准机关 （盖章）

国家食品药品监督管理局培训中心

培训证书

王振迪 同志于 2009 年 7 月 4 日至

2009 年 7 月 7 日参加 药物临床试验

质量管理规范（GCP）培训班 学习

完成培训计划全部课程。

特发此证

学时：32

学分：10

2009 年 7 月 [illegible] 日

国家食品药品监督管理局培训中心
培训专用章

证书编号：20091062[illegible]

11. 引言

11.1 研究背景

1.1.2 基本概况

为扩大移植供肾池，特殊供肾逐渐被应用于临床并讨论其疗效，其中小儿双肾移植历经50余年的发展，不断突破体重的限制，表现出了令人期待的疗效。[1]在中国，婴儿死亡率约为1.393%，考虑到庞大的人口基数，倘若将此类供肾成功应用于临床将会有效缓解供肾短缺的现状。[2-3]然而世界范围内，较高的技术要求、较多的围手术期并发症以及远期预后的担忧，大多移植中心对低体重小儿双供肾移植仍持谨慎态度，其中，体重小于5Kg的小儿双肾移植更是鲜有应用。[4-5]

Wijetunga等[6]对比分析了15例体重小于5 kg供者与15例体重介于5 kg-20Kg供者双肾移植后效果，两组移植肾一年生存率分别为86.7%和93.3% （P = 0.85）。尽管小于5 kg供者供肾功能恢复期更长，早期移植物丢失的风险高，但仍是值得期待的供肾来源。

11.2 研究类型

观察性研究

回顾性分析成人尿毒症患者移植体重小于 5Kg 新生儿双肾术后疗效

11.3 研究依据

已有文献报道了小儿双肾移植优于活体肾移植的远期术后疗效，由于复杂的操作要求，尚无系统的经验及疗效可以借鉴。本研究通过总结小于5kg供者双肾移植的疗效，并分析了术后并发症的风险因素，探究低体重供者肾脏移植的可行性并推广应用。

11.4 风险/获益评估

11.4.1 已知的潜在风险

本方案为观察性研究，所有手术及治疗均已完成，仅收集病历及随访信息，对患者无干预措施，无操作风险。

受试者存在隐私泄露风险，在病历信息以及随访信息收集过程中，可能因采集人大意、存储数据被剽窃、盗取等的可能，相关供、受者的基本信息以及手术数据泄露。

针对已实施的小儿双肾移植，有文献报道术后移植肾失功、血栓、移植肾功能延迟恢复、急性排斥反应、漏尿等并发症的发生风险可能增加。该类器官属于临床已批准供肾来源。

11.4.2 伤害的概率

该研究不会对患者产生伤害，有罕见可能产生受试者信息泄露。

11.4.3 伤害的程度

被观察患者不会受到操作性伤害，若发生信息泄露，有轻度伤害受试者身心的可能，无致死性不良事件。

11.4.4 已知的潜在获益

该研究回顾性分析手术患者近远期疗效，通过完善病程对患者本人的后续诊疗产生获益。主要研究人员为受试者的直接手术医生，患者后续随访依然需要手术医生的诊疗。在多次熟悉了解患者既往病史的条件下，相关诊疗措施会更精确；

该研究总结相关手术预后及经验，对该类手术后续实施有益，即通过总结分析已有数据，判断出该手术的风险因素，在后续治疗中减少患者暴露风险的可能；

针对手术本身，已有大量研究表明肾移植较规律透析能明显改善尿毒症患者生存率，此类手术的实施扩大了供肾来源，对实施手术的患者有益；同时，长期存活的小儿双供肾受者可能有优于亲属肾移植受者的移植肾生存时间。

11.4.5 潜在风险/获益评估

该观察研究需定期审查数据收集的可靠性与安全性，定期检查数据存储的可靠性，评估信息泄露风险。在数据分析整理后，受试者即可有病情诊疗的获益。

11.4.6 讨论

小儿供肾的应用作为扩大供肾来源的有效方式已得到广泛认可。对于体重小于5Kg新生儿双供肾移植，由于较高的技术难度要求，相关报道少。从已知经验得知，体重小于5Kg新生儿双供肾移植可能增加术后并发症的发生风险，然而与透析相比，仍能极大地改善尿毒症患者生存率。在供肾短缺的背景下，是扩大供肾来源的有效方式。

本观察研究仅收集病历系统以及国家随访系统的数据，不会对患者产生风险，此外，患者通过系统回顾，可能对后续病情治疗有益，且研究结论有益于此类手术的继续开展。

12. 研究目的/终点

主要目的：总结分析近远期移植物生存率，评估体重小于5Kg小儿双供肾的可行性。

次要目的：通过总结分析移植术后并发症发生率及移植肾生长发育情况，了解该类供肾的安全性及有效性。

13. 研究设计

13.1 总体设计

该研究为观察性研究的病例回顾性研究，回顾性分析既往开展该类手术的相关临床数据并总结经验

该研究共计 4 名研究人员负责收集数据，数据来源于 2 部分：既往住院病历系统以及国家肾移植患者随访系统。数据收集标准详见病例信息采集表，采集后由专人整理归档分析。所有数据收集与整理对研究外人员保密。

13.2 样本量

总结开展此类手术至今所有的移植数据共计42例。

14. 研究人群

14.1 诊断标准

供者：体重小于 5Kg 小儿供者，供者入选标准符合我国公民逝世后器官捐献标准。

受者：尿毒症患者

14.2 入选标准

因肾脏原发病、遗传病、代谢、系统性疾病及梗阻导致的尿毒症患者，在明确告知供者类型及手术风险后接受手术者。

14.3 排除标准

小儿双供肾受者的筛选上我们在应用常规肾移植排除标准的条件下还遵循以下排除标准:(1)顽固性高血压及长期未控制的糖尿病、冠心病史或外周血管疾病史、高凝状态、尿路异常；(2)PRA>10%、淋巴毒实验阳性、二次/多次移植、红斑狼疮；(3)既往依从性差。

15. 评价

15.1 主要及次要终点/结局评价

主要终点：近远期移植物生存率，肾移植受者生存率。

次要终点：术后并发症发生率（移植物功能延迟恢复、排斥反应、出血、尿路并发等）、移植肾生长情况以及肾功能恢复情况。

15.2 安全性评价

通过将肾移植受者生存率数据与已发表的尿毒症患者生存率进行对比。

16. 不良事件与严重不良事件

该研究为观察研究，不良事件包括数据收集错误，数据泄露，数据分析错误。

对于手术本身，不良事件：移植术后并发症发生，例如功能延迟恢复、排斥反应、出血、漏尿等术后并发症的发生

严重不良事件：移植肾失功、移植受者死亡

17. 统计分析与统计方法

使用 SPSS 22 版和 Windows Origin 9.00 版进行所有统计分析。所有计量资料以均数和标准差表示。Kaplan - Meier 生存曲线用于评估移植物存活率的差异。计量资料采用卡方检验，2 个独立样本比较采用 t 检验，结果以 $P<0.05$ 为差异有统计学意义。

18. 受试者的医疗与保护

18.1 受试者在研究中风险的评估以及风险处置措施、预案

数据收集错误与数据分析错误

评估、处置措施：研究共计 4 人收集数据，每一份数据至少有 2 次收集后核对，当数据收集完毕后会统一评估数据的离散度，在发现明显异常数据后追溯原数据，同时修改。

数据泄露

评估、处置措施：所有数据收集后均统一保存于 1 台电脑及一份电子备份，数据文件夹设置密码，仅供本研究人员使用。

19. 支持性文件与注意事项

19.1 隐私保护

本研究所有资料仅研究申请人及参与人能够获得，已对参与者明确告知对供者和受者的信息保护。

19.2 标本、资料的收集与使用

该研究不存在第三方使用数据，所有数据仅供该研究使用。

19.3 质量控制与质量保证

1.该供肾种类得到了我国器官获取组织的批准，是合法的供肾来源之一；

2.该手术方式早期已通过我院新技术新业务申报的批准；

3.通过我院器官移植伦理委员会的审批；

4.严格数据收集标准，4 人收集数据相互比对后得到最终回顾性分析结果；

5.移植术后随访数据专人管理，定期记录患者肌酐恢复等情况，同时数据与国家系统保持一致，确保数据真实性与可靠性。

19.4 数据处理与记录保存

19.4.1 数据收集与管理

整理患者既往病历，收集拟研究的相关数据及预后情况；长期随访数据通过登录中国器官移植随访登记系统查询。对于每一位肾移植术后患者，随访数据均要求专人上传于系统中，在本研究中，共计3人参与了数据的收集与整理，并相互核查数据录入是否有误。

19.4.2 研究数据保留

已将所有病历数据通过图片形式保存于电脑中，患者随访数据储存于中国器官移植随访登记系统中。

19.5 利益冲突声明

已签署利益冲突声明，见附件。

20. 参考文献

[1] Pelletier S J, Guidinger M K, Merion R M, et al. Recovery and utilization of deceased donor kidneys from small pediatric donors[J]. Am J Transplant, 2006,6(7):1646-1652.

[2] Meakins J L, Smith E J, Alexander J W. En bloc transplantation of both kidneys from pediatric donors into adult patients[J]. Surgery, 1972,71(1):72-75.

[3] Dai H, Peng L, Peng F, et al. A novel technique for en bloc kidney transplantation from infant donors with extremely low body weight by using the distal abdominal aorta as an outflow tract[J]. Am J Transplant, 2018,18(9):2200-2207.

[4] Wijetunga I, Pandanaboyana S, Farid S G, et al. Neonatal kidney donation and transplantation: a realistic strategy for the treatment of end-stage renal disease[J]. Arch Dis Child Fetal Neonatal Ed, 2014,99(6):F518-F519.

[5] Mohanka R, Basu A, Shapiro R, et al. Single versus en bloc kidney transplantation from pediatric donors less than or equal to 15 kg[J]. Transplantation, 2008,86(2):264-268.

[6] Wijetunga I, Ecuyer C, Martinez-Lopez S, et al. Renal transplant from infant and neonatal donors is a feasible option for the treatment of end-stage renal disease but is associated with increased early graft loss[J]. Am J Transplant, 018,18(11):2679-2688.
