# Supplementary material for: En-Bloc Kidney Transplantation From Extremely Low-Weight (0.9–5.0 kg) Pediatric Donors: A Decade of Single-Center Experience
Source: Transpl Int. 2025 May 20;38:14451. doi: 10.3389/ti.2025.14451 (PMC12131009; doi:10.3389/ti.2025.14451)
Supplement: Supplementary file 6 [file DataSheet5.PDF]

Supplementary file 5 Indicative biopsy results of recipients two years after renal transplantation

	Days after transplantation	Proteinuria	Pharmacological agents for reducing urinary protein levels	Banff score	Glomerulosclerosis	Pathological diagnosis
1	1092	2+	Yes	i1,t0,g0,v0,ci0,ct1,cg0,cv0,mm0,ah0,ptc2,C4d3	0/36	ABMR
2	1007	-	No	i1,t0,g0,v0,ci1,ct1,cg0,cv0,mm0,ah2,ptc0,C4d0	2/25	Possible recurrence of IgA
3	907	+	Yes	i0,t0,g0,v0,ci0,ct0,cg0,cv0,mm0,ah0,ptc0,C4d0	0/11	IgA
4	605	-	No	i0,t0,g0,v0,ci0,ct0,cg0,cv0,mm0,ah0,ptc0,C4d0	1/16	CNI nephrotoxicity
5	558	-	No	i3,t2,g1,v1,ci1,ct1,cg0,cv0,mm0,ah0,ptc2, c4d0	0/19	AR, IgA
